# Analysis of Movement Entropy during Community Dance Programs for People with Parkinson’s Disease and Older Adults: A Cohort Study

**DOI:** 10.3390/ijerph19020655

**Published:** 2022-01-07

**Authors:** Peter Gates, Fred M. Discenzo, Jin Hyun Kim, Zachary Lemke, Joan Meggitt, Angela L. Ridgel

**Affiliations:** 1 Program of Exercise Physiology, Kent State University, Kent, OH 44240, USA; pgeczy@kent.edu (P.G.); phdjhk60@gmail.com (J.H.K.); zlemke@kent.edu (Z.L.); 2 Rockwell Automation, Mayfield Heights, OH 44124, USA; fmdiscenzo@gmail.com; 3 Theater and Dance, Cleveland State University, Cleveland, OH 44115, USA; j.meggitt@csuohio.edu

**Keywords:** movement disorders, rehabilitation, kinematic analysis, Parkinson’s dance, dance analysis, tango, time series analysis

## Abstract

Dance therapy can improve motor skills, balance, posture, and gait in people diagnosed with Parkinson’s disease (PD) and healthy older adults (OA). It is not clear how specific movement patterns during dance promote these benefits. The purpose of this cohort study was to identify differences and complexity in dance movement patterns among different dance styles for PD and OA participants in community dance programs using approximate entropy (ApEn) analysis. The hypothesis was that PD participants will show greater ApEn during dance than OA participants and that the unique dance style of tango with more pronounced foot technique and sharp direction changes will show greater ApEn than smoother dance types such as foxtrot and waltz characterized by gradual changes in direction and gliding movement with rise and fall. Individuals participated in one-hour community dance classes. Movement data were captured using porTable 3D motion capture sensors attached to the arms, torso and legs. Classes were also video recorded to assist in analyzing the dance steps. Movement patterns were captured and ApEn was calculated to quantify the complexity of movements. Participants with PD had greater ApEn in right knee flexion during dance movements than left knee flexion (*p* = 0.02), greater ApEn of right than left hip flexion (*p* = 0.05), and greater left hip rotation than right (*p* = 0.03). There was no significant difference in ApEn of body movements (*p* > 0.4) or mean body movements (*p* > 0.3) at any body-segment in OA. ApEn analysis is valuable for quantifying the degree of control and predictability of dance movements and could be used as another tool to assess the movement control of dancers and aid in the development of dance therapies.

## 1. Introduction

Parkinson’s disease (PD) is a chronic condition marked by the degeneration of dopaminergic motor neurons in the substantia nigra pars compacta of the midbrain [1,2]. The resulting dopamine deficiency leads to the identifiable motor symptoms of PD including slow movement (bradykinesia), tremors at rest, and rigidity of the upper and lower limbs [2]. Nonmotor symptoms include cognitive decline, sensory and sleep abnormalities, dementia, fatigue, depression, and anxiety disorders [3,4]. Over two thirds of individuals with PD also see loss of some autonomic nervous system control [5]. By the time motor symptoms are identifiable, up to 70% of dopaminergic neurons in the substantia nigra have degenerated [6]. There were approximately 680,000 individuals aged 45+ in the US afflicted with PD in 2010 and this is projected to rise to 1,238,000 by 2030 [7].

Exercise reduces the risk of falls [8,9], and improves mobility [9,10,11], body composition [12], sleep quality [13], and depression [14,15,16] in older adults. Individuals with PD showed improvements in bradykinesia [17,18,19], tremors [20], and overall Unified Parkinson’s Disease Score Part III: Motor Examination (UPDRS III) [21,22,23], a measure of PD motor symptoms. Walking, cycling, and weight training are the primary exercise modes examined in research trials. Specifically, high-cadence cycling promotes improvements in UPDRS Motor III scores, bradykinesia, and tremors in individuals with PD [18,20,24,25] and the driver of these improvements is proposed to be the variability (entropy) of cadence. Other movement-based activities such as Tai-Chi [26] and dance are also beneficial in these populations. Dance has previously been shown to improve the motor and cognitive symptoms of PD including UPDRS III [27], balance [28], gait [29], executive function [30], and depression [31]. Specifically, several studies in PD have shown that tango improves motor symptoms, balance and gait more than other types of dance [28,32]. The mechanisms behind these improvements and the biomechanical characteristics of movements that drive the benefits are unknown. However, it has been proposed that a potential driver of the motor function improvements from tango dancing may be due to the slow and quick movement steps occurring primarily forward and backward, and the need for more precise foot speed and placement [33]. Additionally, tango is danced with a particularly closed dance hold, firm posture and with strong movement cues from the partner. Lastly, the music typically has a more rhythmically clear strong downbeat on the first beat of a measure and sharper corners when changing direction [34].

The “loss of complexity” hypothesis states that variance in physiological processes decreases with age, resulting in decreased ability to adapt to stressors and thus increasing risk of injury or disease [35]. Therefore, therapies that incorporate complex movements that improve the ability to respond to unexpected stressors could offset this “loss of complexity”. Approximate entropy (ApEn) is a measure of the regularity of data in time series [36]. It takes as input the length of the dataset (N), a tolerance of similarity (r, usually 0.2 * the standard deviation), the length of compared pattern (m, usually 2), and the delay number (tau) or the smallest lag number at which the autocorrelation function (ACF) is closest to 0. The ACF is a correlational measure of a time series with itself, at a prespecified delay. A delay of 100 indicates that each value A in the time series was correlated with a value B that is exactly 100 values away. The delay number at which ACF is lowest, termed tau, thus represents the minimum distance B must be for the lowest correlational coefficient. If a time series is correlated with itself where the lag is equal to tau, the correlation is very weak and relatively unpredictable. Using this value of tau, it is possible to determine ApEn and quantify disorder or predictability within the time series. The magnitude of values in the dataset does not affect the result of ApEn, which is closer to 0 if the dataset has increased regularity or decreased disorder.

There are several examples in the literature where ApEn of movement decreases with lack of automaticity. When focus is directed solely to balance, participants experience a decrease in ApEn of balance compared to balancing while focusing on an external task [37,38]. In addition, the ApEn of flexion-extension of an anterior cruciate ligament-deficient knee while walking is smaller than a healthy knee even across different speeds [39]. Parkinsonian tremors have decreased ApEn than physiological tremors [40,41]. In a unique analysis of the spiral drawing task, the ApEn was used to calculate a new score called the “temporal irregularity score”, which decreased with more advanced stages of PD [42]. Voluntary wrist extension and flexion in participants with PD was also seen to have decreased ApEn as compared to healthy older adults [43]. Tandem cycling also showed increased ApEn of cadence as a key indicator of improvements in PD [25,44,45]. These examples illustrate a unique relationship between ApEn and PD symptoms and motor function and provide a potential avenue for dance movement analysis.

Previous studies of dance and forced exercise provided compelling evidence that movement and rhythmic dance can provide improvements in standardized performance tests for people diagnosed with Parkinson’s disease [46,47,48]. The benefits of dance observed for people with Parkinson’s include motor and non-motor skills and these benefits are also realized by older adults [49,50]. These studies, often performed in a laboratory setting, are typically observational and focused on assessing the impact of the dance or exercise intervention without consideration for the specific steps or movement characteristics of the participants during dance. The goal of this study was to demonstrate a protocol for studying differences in biomechanical movements of PD and older adults (OA) that may provide a basis for the design of dance programs targeted at improving the motor skills of participants with PD and healthy older adults.

## 2. Materials and Methods

### 2.1. Study Design

This was a cohort study where high-resolution movement data from PD and OA dancers were captured during dancing in PD and OA-specific community dance classes. Captured data during dance were analyzed with the objective of identifying underlying patterns or movement characteristics. The statistical characterization of PD and OA dance movement provides a useful tool for analyzing dancer motor skills and a foundation for directing future dance or exercise movement to further improve the motor skills of PD and OA participants. The study phases were:

1. Study design, institutional approvals, preparation and equipment calibration;
2. Solicitation of PD and OA dance volunteers at the dance site;
3. Acquisition of dynamic data during dance;
4. Data validation and time-based correlation with video captured during class;
5. Extraction of statistically relevant movement features for PD and OA participants;
6. Identification of significant movement features related to motor skill performance;
7. Establishment of a framework to define future movement patterns that will improve critical movement features to optimize motor skill performance.

### 2.2. Ethical Considerations

Prior to any project work or data collection, the study design, testing protocol and informed consent was submitted to Kent State University’s IRB for review and approval (Application Number 18-248). All ethical standards including informed consent, minimizing risk to the dancers, protection of their anonymity and confidentiality and freely (uncoerced) choice to participate or withdraw from the study.

PD dance classes provided by Come Dance With Me were held monthly at a Human Services Center. These classes are free for people diagnosed with Parkinson’s disease and their care partner. The study was described to the entire PD dance class at the beginning of each class by a member of the research team who was not a dance instructor. One participant from each class over a seven-month period was recruited to volunteer for the study. Each dancer freely volunteered to participate in this one-class data capture effort. Volunteers provided written informed consent as required by the Institutional Review Board.

OA dance classes were held at Bowling Green State University, Firelands Campus. Three two-hour classes, Dance Your Way to Health and Happiness, were offered to seniors by Bowling Green State University ElderCollege, Office of Educational Outreach. The study IRB 18-248 approved by Kent State University was also approved by Bowling Green State University as meeting their IRB requirements. Similar to the PD classes, any registered class participant was free to participate in the study without coercion. No grades are given and there were no consequences for seniors to participate or withdraw from the study. The study was described to the entire class at the beginning of each class and two volunteers were recruited per session, for one hour of observation time each.

The same instructors and co-investigators oversaw dancer training, dancer volunteer recruitment and data acquisition for both the PD and OA dance classes. Non-invasive, strap-on wireless sensors were attached to arms, legs, and torso of the dancer participants so movement would be minimally affected by the instrumentation. All dance instructors were trained in safe movement techniques for seated and standing movement and transition between sitting and standing. PD volunteers were screened to make sure they could safely perform basic dance steps being taught and could participate for the duration of the class without a walker.

### 2.3. Participants and Class Overview

In all cases, both PDd and OAd volunteers had very little to no prior ballroom dance training. With practice, all participants were able to perform the basic ballroom steps taught and stepped with the beat of the music. Ballroom dance technique and style methods were briefly presented (e.g., step size, foot passing, foot placement) but the focus was having the participants perform the dance steps safely to the beat of the music.

Foxtrot, tango and waltz were danced in closed dance position with one of the partners (leader or follower) participating in the study. In partnered PDd classes, one partner (leader or follower) is diagnosed with PD and the other is not. In partnered OAd classes, both leader and follower have no defining motor skill deficits. Line dances were performed with free (non-partnered) movement. The PDd and OAd dance classes included line dances and partnered ballroom dancing. The classes included waltz, foxtrot, tango, and swing. Additionally, the PDd classes included rumba and cha cha, and some tango was also performed in every class in recognition of the published benefits of tango for PD dancers [28,30,31,32,33]. Ballroom dancing was performed partnered and mostly in the closed partner dance position although while learning the steps, an open two-handed hand-to-hand partner position was used. Line dances were performed in a free, no hand holding, position. Ballroom bronze-level school figures were taught in both the PDd and OAd classes that included basic box steps, progressive steps, hesitation steps and under arm turns. Tango dance was taught using standard American tango steps including basic progressive, corté and promenade. Practice was performed to popular music with a strong beat and the tempo was adjusted as needed to ensure students could perform the dance steps safely and properly to music. Music volume was slightly higher than usual to help the dancers hear the music beat and to accommodate the senior dancers (both PD and OA) with hearing deficiencies. The same instructors were used for both the PDd and OAd classes and they have been trained in dance instruction for PD (Dance for PD^®^ [51]) and ballroom dancing (ballroom silver medal certification [52]).

### 2.4. Data Acquisition

**Figure 1:**
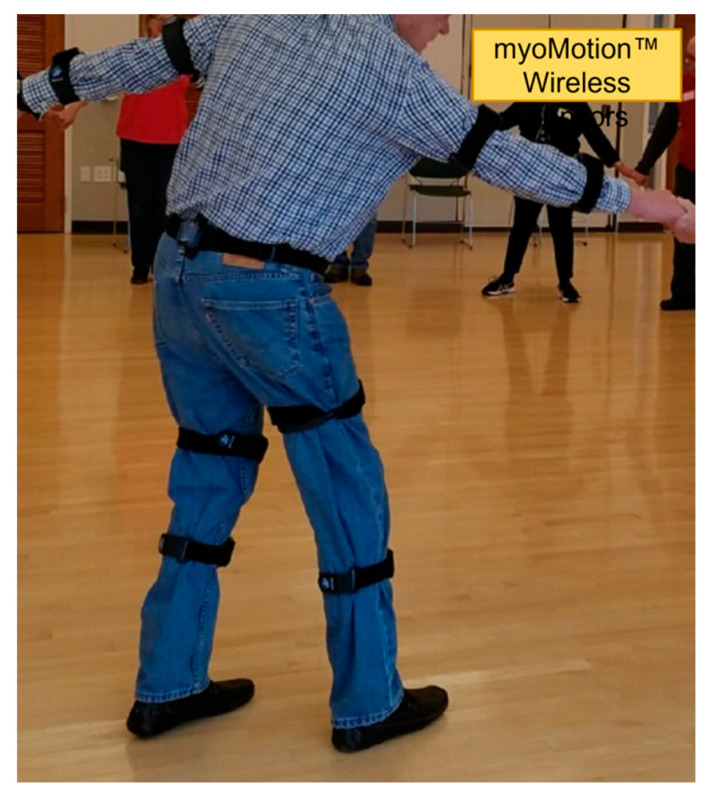
Dance students wore the Noraxon myoMOTION™ devices for a one-hour long session. These devices were attached to the arm, forearm, hip, thigh, and leg, and activated during each dance practice.

Noraxon myoMOTION™ [53] hardware (Noraxon USA, Scottsdale, AZ, USA) was used to capture timeseries movement data by attaching motion sensors to the upper arm, forearm, pelvis, thigh, and leg on both sides of each participant (Figure 1). These measured the acceleration, velocity, orientation, and joint angle movements of students during community-based group dance classes. Prior to data capture, a calibration procedure was performed for each test subject to ensure proper system operation and to permit data capture from each sensor in a common reference frame. Motion was captured each time the lesson for a step ended and practice began, marked by the start of music, and not limited to any period within the class.

**Figure 2:**
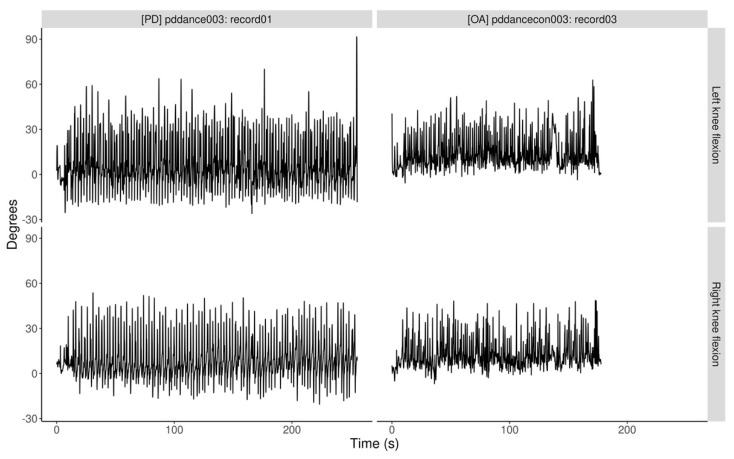
Tango: left and right knee flexion over time. PD (**left**) and OA (**right**) recordings of left and right knee flexion during one session of tango for one PD and one OA participant, no filtering or down sampling was performed.

The frequency of data capture was set at the equipment’s default of 100 Hz. No application filtering or downsizing of the time series was performed. Data were analyzed as received from the myoMOTION software (Version 3.12). The captured data included joint movement (deg) of the left and right elbow, hip, and knee; body orientation on x, y, z, w axis; trajectories (mm); and body segments. In addition, time-stamped video recordings were made during each of the dance classes. This helped in selecting the appropriate section of the captured time series data (Figure 2) for analysis and aided in interpreting the data in context of the activity being performed.

### 2.5. Study Procedure

Six major dance types, tango, waltz, foxtrot, line, rumba, and swing were recorded. There were only two recordings of rumba and one of swing, both from PD participants, so these were removed from analysis. Previous research shows that Argentine tango is more effective at motor symptom management in PD [27,31,33,54,55,56] than waltz, foxtrot, or Tai Chi for example, therefore this paper’s focus is analyzing motion during tango. All participants in PDd and OAd classes danced American (also called ballroom) tango. However, due to the significant similarity in the music and dance style with Argentine tango and American tango it is appropriate to consider the participants movements in these classes while doing American tango. American tango music is characterized by a strong, pronounced steady beat with a strong downbeat at the beginning of each measure and dancers use strong body movement during progression around the dance floor using an eight-count sequence of dance steps. Due to equipment error many of the upper limb variables were not recorded during several test sessions. Therefore, this study focused on analyzing lower limb measurements of the pelvis, thigh, and leg. This does not cause a problem since most of the motion and technique for tango as well as the other dances studied occurs from the waist down and changes in balance, posture and gait will be primarily affected by lower body movement.

### 2.6. Data Analysis

**Figure 3:**
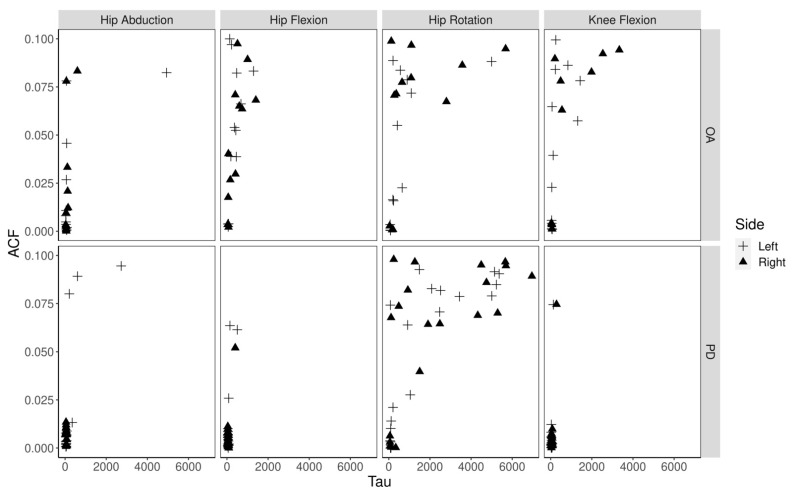
Tau values at each limb measure. An unconventional ACF plot showing the ACF values closest to 0 at the lowest possible tau (lag). Each recording’s observed tau values were used to calculate the respective ApEn. While there was no statistical significance in overall left vs. right and overall PD vs. OA tau values, the values for knee flexion in OA has a much wider range than in PD. The ApEn of knee flexion was also found to be significantly lower in left knee flexion than right knee flexion in PD (t(12.5) = 0.63, *p* = 0.02), however in OA there was no difference (t(7.0) = 0.10, *p* = 0.93).

The approximate entropy (ApEn) of joint movement was calculated using the MatLab script previously created for analyzing data from the dynamic bike study [45] conducted by several authors of this paper. An overview of the ApEn algorithm is provided as Supplementary Materials File S1. The MatLab script was modified to take dance time series as input, but the calculation of entropy was unchanged. The delay number parameter, tau, was calculated by using the smallest lag for each recording at which ACF was closest to 0 (Figure 3), in accordance with findings that these estimated delay numbers better capture the entropic nature of time series data [57,58].

Following ApEn calculation of joint movement recordings, results were grouped and averaged by participant, left or right limb movement, and dance type. This was performed as some participants had multiple recordings of the same dance type. Finally, descriptive and mean statistical tests were run separately on both datasets. The hypothesis that there is a significant difference in left and right approximate entropy of movement in PD but not in OA participants was tested with two-sided Student t-test using the default t.test function in the R [59] language. The rstatix [60] library for R was used to test for outliers, equality of variance, and the normality assumption. The ggplot [61] and ggpubr [62] libraries were used for graphing.

## 3. Results

A total of 7 PD (all male; 69 +/− 7.5 years; body mass index (BMI) 26.4 +/− 3.8) and 5 OA (3 female, 2 male; 73 +/− 4.6 years; BMI 25.3 +/− 4.4) participated in this study. A total of 31 dance recordings were captured for analysis (20 PD dance recordings, 11 OA dance recordings). As shown in Table 1, some of the study subjects participated in multiple dance types and some had multiple data capture sessions of the same dance type. Data from one individual with PD was removed from the dataset due to invalid or incomplete time series data from malfunctioned equipment. There was no significant difference in BMI (t(7.86) = −0.41, *p* = 0.69) or age (t(6.68) = 1.02, *p* = 0.35) between the two groups. The mean length of recorded sessions for PD group was 2.1 +/− 1.1 min and 3.5 +/− 1.4 min for OA, but this difference was not significant (t(9.19) = 2.06, *p* = 0.07). Table 1 shows mean session time and number of recordings per dance type. Tango was the most prevalent dance session captured for analysis in the PD group with 13 recordings from five participants. While the PD group had more recordings, the OA group had longer recordings.

**Figure 4:**
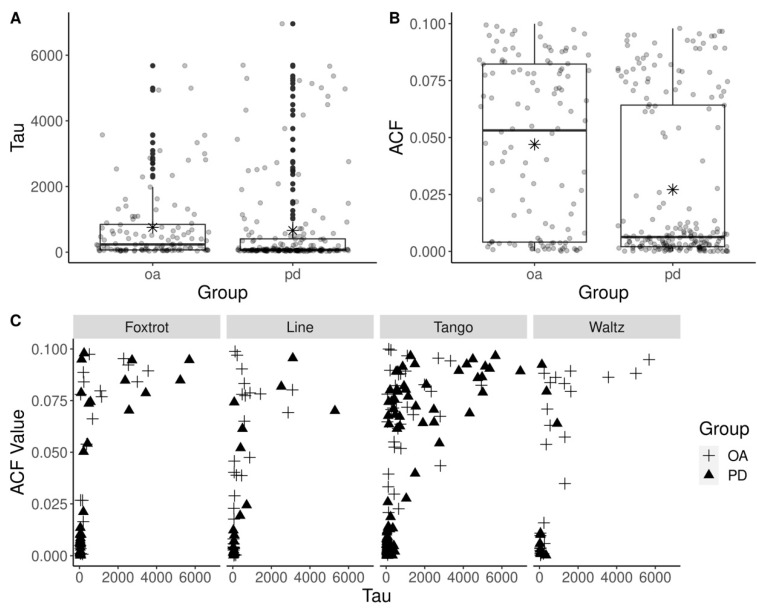
Delay numbers used for ApEn calculation (**A**). ACF closest to 0 at the smallest lag value (tau) (**B**). Tau values at each dance category (**C**). Datapoints include tau values for hip and knee movements. Distribution of delay numbers (OA 753.72 +/− 1130.05; PD 660.49 +/− 1373.47) used for ApEn calculation (**A**) and their correspondent autocorrelation functions (OA 0.047 +/− 0.036; PD 0.027 +/− 0.035) (**B**). Delay numbers were calculated by limiting ACF to < 0.1 coupled with 100 Hz sampling explains the large values for lag. Asterisk (*) represents the mean. C presents tau values per dance and group where one data point is one recording. These were used to calculate ApEn for each recording. No significant difference in tau values were found between PD and OA (*p* = 0.89) or between dance groups (*p* = 0.88).

The mean recording length was not significantly different between PD and OA (t(9.19) = 2.06, *p* = 0.07) or between dance categories (F(3, 13) = 0.18, *p* = 0.91). To calculate the ApEn of left and right joint movements, the smallest lag number for the ACF closest to 0, (limited to < 0.1) was used. These values were much larger (Figure 4A) than the typically used delay numbers ranging from 1 through 5, however given the high frequency (100 Hz) of measurements, the previously mentioned findings, and the predefined limit on max ACF, these lag values were found acceptable and used in the ApEn calculation. The median ACF at which tau could be defined in the PD group was closer to 0 than in the OA group (Figure 4B), and the difference in means was significant (t(10.22) = 3.18, *p* = 0.01). The calculated lowest tau for each recording per dance type is shown in Figure 4C. The lowest tau was used for ApEn calculation.

**Figure 5:**
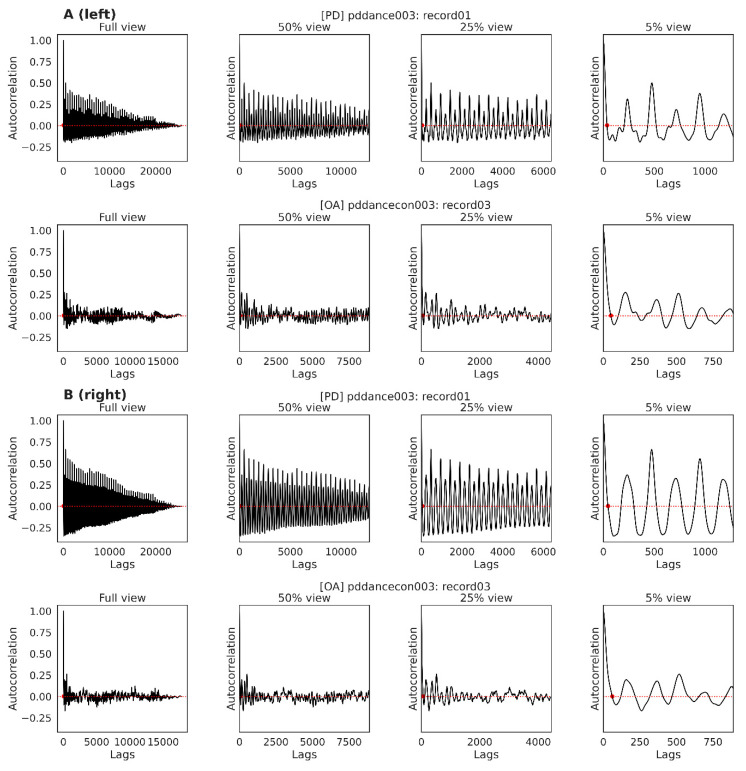
**Left and right knee flexion during tango.** Demonstrating ACF plots of left (**A**) and right (**B**) knee flexion for one PD and one OA participant. Red dot represents the selected lag value (smallest lag value where the ACF is closest to 0). The graph represents correlations of a time series with a lagged version itself. A lag of 1 indicates correlating time series with itself, which will be perfect correlation. Lag of 2 indicates correlating time series with a version of itself that was shifted to the right by one time unit. Possible number of time units is the length of the time series.

There was no significant difference in mean tau between the dance types (F(3, 14) = 0.22, *p* = 0.88) or between the PD and OA groups (t(15.99) = 0.14, *p* = 0.89). Figure 5A provides example ACF plots for left knee flexion for a PD and OA participant, and Figure 5B for right knee flexion, during Tango for the same PD and OA participant. The ACF was calculated for lag values in range of the length of the recorded dataset.

**Figure 6:**
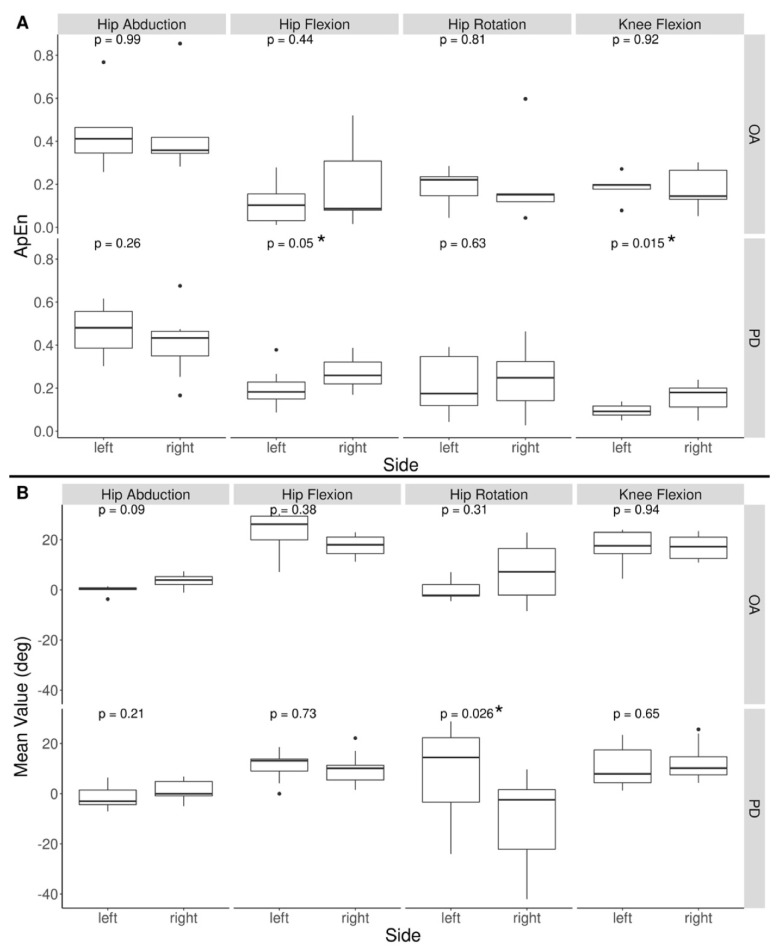
Left vs. right ApEn of body movement in PD and OA (**A**). Boxplot of ApEn values where each datapoint represents the mean ApEn of all recordings for a specific dance and specific participant. No significant difference was found between left and right ApEn of movement in the OA group. The PD group had higher ApEn of right knee flexion and hip flexion than left. Left vs. right mean body movement in PD and OA (**B**). No significant difference was found between mean left and mean right body movements in OA, however PD saw a significantly higher left hip rotation than right. * indicates measurements with a significant difference in ApEn between left and right knee flexion and hip flexion for PD participants, and a significant difference in mean left and right hip rotation for PD participants.

ApEn of joint movement in the PD group passed the Shapiro–Wilk’s test of normality (*p* > 0.1 at all joints), Levene’s test of equality of variance (*p* > 0.1 at all joints), and no extreme outliers were found. Right hip abduction and right hip rotation in the OA group’s ApEn results did not pass Shapiro’s test (*p* < 0.05), so these variables were analyzed using the Mann–Whitney test. All variables passed Levene’s test (*p* > 0.4 at all joints). One outlier in right hip abduction, one in right hip rotation, and two in left knee flexion were found. These values were kept due to the small sample size. There was a significant difference (Figure 6A) between ApEn of left and right knee flexion (t(12.49) = −2.68, *p* = 0.02) with hip flexion close to significance (t(17.88) = −2.10, *p* = 0.051) in the PD group. There was also a significant difference in the mean left and right hip rotation of PD (t(16) = 2.46, *p* = 0.03) but not OA (Figure 6B). OA did not see any significant difference in the ApEn of left and right body movements. No other significant difference in the means of left and right body movement was found in either group.

## 4. Discussion

The goal of this study was to identify methods for quantifying and analyzing the dance movements of PD and OA participants in community dance programs. The intent is for this analysis to provide a basis for selecting or designing effective dance programs for PD or OA participants in the future.

To calculate the ApEn of each recording, tau, defined as the lowest delay number at which ACF is closest to zero, had to be identified. The value for tau was not only much larger than the expected values between one and five, but the range of tau was wider as well. While there was no significant difference in tau between PD and OA groups, there was a difference in the lowest ACF at which tau was found. Since the goal was to find the tau at which ACF is closest to 0, and ACF is a correlational measure of a time series with itself, any ACF < 0.1 was assumed to satisfy the goal. Due to the frequency of data capture (100 Hz) and the small participant population size, it is perhaps not surprising that the magnitude of tau was so large. Further research is needed to determine whether tau values change with activity type, such as during dynamic biking or dancing, and to confirm previous findings [57] on its relationship to ApEn in the context of physical movement.

ApEn was shown to be a useful measurement in analyzing movement patterns and, as only the PD group showed any difference between left and right sided ApEn of movement, that the “loss of complexity” hypothesis may extend to dance movements as well. All participants with PD reported higher intensity of symptom presentation on the left side than the right side. These findings support the idea that as automaticity is lost because of PD progression, the ApEn of movement decreases. This was most apparent in knee flexion and extension. Although body segment acceleration, pitch, course, and roll were not analyzed, it is likely to be affected as well. Previous studies reported improvements in the presentation of PD symptoms following sessions of Argentine tango [27,30,31,33,54,55] through an unknown mechanism. In previous studies examining the effects of high-cadence cycling on symptom presentation of PD, entropy was found to be a key factor associated in observed improvements in movement [18,24,44,45]. This variance, measured both as ApEn and a similar measure called sample entropy, is thought to be a function of the trainer’s (captain) high cadence and the stoker’s (participant with PD) efforts to keep up with that cadence. As the participant strives to match the trainer’s cadence ApEn is introduced [63]. Partnered dance has similar interplay, where PD and non-PD subjects are paired up to carry out predefined movements. In this way, tango might introduce increased entropy to the participant with PD and thereby share a similar but different mechanism as documented in the tandem bike studies.

Patients with Parkinson’s disease or Huntington’s disease have been shown to have depressed proprioception-related potentials [64]. It is believed that proprioception deficits affect sensorimotor integration used for postural control and limb movements. Furthermore, the degree of proprioception deficits correlates with the severity of Parkinson’s disease. The resulting kinesthesia (awareness of body or limb position and movement in space) likely affected the timing, accuracy, and reproducibility of dance movements for PD dancers in this study. The use of ApEn can serve as a surrogate suggesting the degree and type of body or limb movement most affected by proprioception sensorimotor deficiency.

It is significant that movement training focused on somatosensory feedback can train and improve proprioception function in patients with mild to moderate Parkinson’s disease [65]. Improvement observed was localized only in the trained joint movement. This suggests that somatosensory feedback training in problematic joint movements such as knee flexion may be an effective technique to improve movement (and improve ApEn). Dance types and dance steps that focus on somatosensory feedback training may be used to improve proprioception function in Parkinson’s dancers.

For example, in this study it was observed that the PD participants exhibited an asymmetric ApEn in knee flexion and hip rotation and possibly hip abduction. In response to this observation a dance regimen consisting of primarily basic tango steps forward and backward can serve to provide proprioception training for hip and knee flexion and ultimately improve ApEn measurements to more closely match that observed for the OA dancers. Similarly, a dance program consisting primarily of basic merengue steps can serve to potentially improve movement accuracy and ApEn for hip abduction. ApEn along with other established Parkinson’s movement assessment protocols may be effective in not only monitoring the effectiveness of a dance program but also to alter a dance intervention program to optimize the improvement in motor skills of Parkinson’s participants.

This study compares general joint movement during dance, not specific to any dance type and indicates a new avenue for future research. A repeated measures study design, where the movements of OA and PD participants recorded across several weekly or twice weekly sessions will provide additional insights and more consistent results. If the finding of decreased entropy on the most affected side holds true it is also likely that ApEn will increase as participants experience improvements in symptom presentation. Due to the social nature of dance, a comparison of ApEn between the participant with PD and their non-PD partner during tango may also provide clues to the mechanism behind observed improvements in other studies.

Future studies focused on the differences in dance movements between PD and OA groups need to be conducted with more comparable dance classes that can control for dance types and movements. As genders perform different roles in dance, gender difference in the groups is likely to have some influence on this study’s outcome. No direct comparison was made between the PD and OA since the two dance groups were not similar: the OA group attended three two-hour dance sessions over a five-week period, while the PD dance group met monthly for one hour. While both groups had the same instructors, the music selection for the PD class often had a much stronger beat and the tempo was often slightly slower to insure a successful dance experience for the PD dancers. It is also important to note that each participant in the PD group was paired with the non-PD spouse or a non-PD volunteer. Participants in the OA group were often with their spouse or a volunteer and not necessarily an OA. The reliance on partners during dancing may influence the measured ApEn of movements.

## 5. Conclusions

This study described the use of multiple, concurrently operating body-worn non-invasive wireless sensors to capture real time, sub-second movement data during dance. High resolution, rapid sampling of movement data provided new insights on PD and OA dancer movement. At the micro-level, analysis provided descriptive statistical measures of dynamic movement (e.g., stepping) during dance and these measures are related to motor skill performance.

A new avenue for future research was demonstrated based on the relationship between several dance styles and dancer performance. For example, the analysis of ApEn highlighted significant differences in left and right sided hip and knee flexion in the PD, but not OA, group. Additionally, the results obtained suggest that ApEn can serve as a surrogate measure of motor skill deficiency that is associated with sensorimotor integration deficits in Parkinson’s dancers. Furthermore, this estimate of motor skill proficiency is a quantitative value that can be used to monitor and track changes in the performance of PD and OA dancers over time.

This study had several weaknesses. First, no definitive conclusions about the dancer performance were made from this analysis due to the small sample size and short duration of the dance session when each dataset was captured (one hour). Motor skill improvement was not expected during a brief dance session, and participant performance was not tracked following the one-hour data capture. Other confounding variables include the condition of the dancer regarding fatigue and fitness level (and stage level for PD dancers), dancer experience, medication state, physical limitations, leader or follower role, and partner interaction. For example, often there is a change in the step pattern for a PD dancer when performing the same dance step with and without a partner. Additionally, some followers provide a very strong lead that greatly influences the movement of their partner. For this initial investigation no consideration was given to the specific dance steps being performed (within a dance type) or music characteristics such as tempo, tempo consistency, clarity of the beat, music rhythms and phrasing, the energy or dynamics of the music or familiarity of the song.

However, the ability to improve proprioception and motor function in PD suggests that a dance regimen focused on improving observed movement deficits, indicated by ApEn measurements provides a basis for designing a dance regimen focused on improving motor skills in PD. The targeted improvement in motor skills is intended to translate into improved postural control, gait and balance. The entropy analysis of movement trajectories during dance promises to provide a basis for assessing and improving motor skills for many OA and PD participants across a wide range of physical abilities and health conditions. Future studies could utilize ApEn analysis of dance movements in PD and OA and determine optimal movement patterns, perhaps with musical cues, to assess performance improvement and maximize benefits that participants with PD.

## Figures and Tables

**Table 1:** Number of participants, recordings, and the mean length of recordings for each dance.

	Foxtrot		Line Dance		Tango		Waltz	
	PD	OA	PD	OA	PD	OA	PD	OA
Participants	2	1	2	2	5	2	1	2
Number of recordings	4	2	2	2	13	4	1	3
Mean session time (min)	1.8	3.2	2.0	4.1	2.3	3.5	1.0	3.0

## Data Availability

The data presented in this study are available on request from the corresponding author. The data are not publicly available due to subject privacy.

## Supplementary Materials

The following supporting information can be downloaded at: https://www.mdpi.com/article/10.3390/ijerph19020655/s1, Supplementary Materials File S1.
