# Supplementary material for: Analysis of Movement Entropy during Community Dance Programs for People with Parkinson’s Disease and Older Adults: A Cohort Study
Source: Int J Environ Res Public Health. 2022 Jan 7;19(2):655. doi: 10.3390/ijerph19020655 (PMC8775546; doi:10.3390/ijerph19020655)
Supplement: Supplementary file 1 [file ijerph-19-00655-s001.zip › ijerph-1534567-supplementary.pdf]

Supplementary Materials

ACF Algorithm: ACF is the correlation of a time series with itself, given a lag. The statsmodels library was used to calculate the ACF at each possible lag value (line 1 in the function). As "lag" is defined as one unit of time in the time series, the total possible lag values is equivalent to the number of data points in the time series. An array of numbers 1-N was created to represent lag (line 2 in the function). The resulting dataframe thus includes ACF after a 1 second lag, ACF after a 2 second lag, and so on up until ACF after a length_of_series lag. This ACF-Lag dataframe is used to find Tau in the next function. The process is repeated for each participant-dance-measurement combination.

```
def get_acf_lag(my_series: pd.Series) -> pd.DataFrame:
  '''
  Finds the ACF at delay numbers ranging from 0 to the length
  of the time series.
  input
  -----
  my_series: the time series for which ACFs should be found
  output
  ------
  new_df:  a  dataframe  containing  each  lag  number  and  its
appropriate ACF
  '''
  # get ACF
  acf = sm.tsa.acf(my_series, nlags=len(my_series), fft=True)
  lag = np.arange(len(my_series))
  new_df = pd.DataFrame({
    'lag':list(lag),
    'acf':list(acf)
  })
  return new_df
```

Tau Algorithm: Tau, the lowest delay number at which the autocorrelational function (ACF) is < 0.1, was found using custom python functions. After calculating the ACF at all possible delay numbers for each participant measure and dance type using the get_acf_lag function, the resulting dataset is run through the find_smallest_lag. It starts off by assuming the lowest ACF is 1 (lines 1-2 of code), which is perfect correlation of the time series with its lag. The dataset is then filtered to include those ACF which are less than or equal to 0.1, and the first 50 rows are extracted (line 3 of code). As this is a time series, the first 50 rows correspond to the first 50 lowest delay numbers. The function then goes row by row to find instances where the lag is less than or equal to 10 and where the ACF is smaller than 1, this is recorded by overwriting the smallest_acf and smallest_lag variables. The next row is then compared to the now overwritten

variable, thereby ensuring that the smallest lag at the smallest ACF is found (lines 4-9 of code).

Figure 5 demonstrates a sample of the results, where the red dot represents tau, the smallest lag with the smallest ACF. The full ACF range is shown on the first graph of each row, with each subsequent plot representing a closer zoom level. Our custom functions found tau at the smallest lag (towards the beginning of the time series delay) and the smallest ACF (closer to 0, representing no correlation between the series and its delayed partner).

```
def find_smallest_lag(dataframe: pd.DataFrame) -> Tuple[float,
float]:
    '''
    Finds the smallest lag where ACF is closest to 0.
    input
    -----
    dataframe: a dataframe of all ACF and accompanying lag values
for a given time series
    output
    ------
    smallest_lag: the first lag at which ACF was <= 0.1
    smallest_acf: the ACF values closest to 0 at the smallest lag
    '''
    smallest_acf = 1
    smallest_lag = 1
    res = dataframe[abs(dataframe['acf']) <= 0.1].head(50)
    for i, row in res.iterrows():
        acf = abs(row['acf'])
        lag = abs(row['lag'])
        if (acf < smallest_acf) & ((abs(smallest_lag - lag) <= 10) |
(smallest_lag == 1)):
            smallest_acf = acf
            smallest_lag = lag
    return smallest_lag, smallest_acf
```
